# Therapeutic Effects of Topical Netrin-4 Inhibits Corneal Neovascularization in Alkali-Burn Rats

**DOI:** 10.1371/journal.pone.0122951

**Published:** 2015-04-08

**Authors:** Yun Han, Yi Shao, Tingting Liu, Yang-Luowa Qu, Wei Li, Zuguo Liu

**Affiliations:** 1 Eye Institute of Xiamen University, Xiamen, Fujian, China; 2 Fujian Provincial Key Laboratory of Ophthalmology and Visual Science, Xiamen, Fujian, China; 3 Department of Ophthalmology, the First Affiliated Hospital of Nanchang University, Nanchang, Jiangxi, China; Children's Hospital Boston, UNITED STATES

## Abstract

Netrins are secreted molecules involved in axon guidance and angiogenesis. However, the role of netrins in the vasculature remains unclear. Netrin-4 and netrin-1 have been found to be either pro- or antiangiogenic factors. Previously, we found that netrin-1 acts as an anti-angiogenic factor in rats by inhibiting alkali burn-induced corneal neovascularization. Here, we further investigate the effects of netrin-4, another member of the same netrin family, on neovascularization *in vitro* and *in vivo*. We found that netrin-4 functions similarly as netrin-1 in angiogenesis. *In vitro* angiogenesis assay shows that netrin-4 affected human umbilical vein endothelial cell (HUVEC) tube formation, viability and proliferation, apoptosis, migration, and invasion in a dose-dependent manner. Netrin-4 was topically applied *in vivo* to alkali-burned rat corneas on day 0 (immediately after injury) and/or day 10 post-injury. Netrin-4 subsequently suppressed and reversed corneal neovascularization. Netrin-4 inhibited corneal epithelial and stromal cell apoptosis, inhibited vascular endothelial growth factor (VEGF), but promoted pigment epithelium-derived factor (PEDF) expression, decreased NK-KB p65 expression, and inhibits neutrophil and macrophage infiltration. These results indicate that netrin-4 shed new light on its potential roles in treatmenting for angiogenic diseases that affect the ocular surface, as well as other tissues.

## Introduction

Netrins are laminin-like secreted molecules that are involved in axon guidance, angiogenesis, and the formation of blood vessel networks [1–4]. The netrin system consists of at least five ligands (netrin-1, -2, -4, -G1a, and -G1b) and six receptors (neogenin, DCC, Unc5A, -B, -C, and -D) [5]. In nervous system, netrins act as bifunctional cues for angiogenesis in axonal guidance. Recently, both netrin-1 and netrin-4 were implicated in angiogenesis [6–11], but the role of netrins in vasculature remains unclear.

Conflicting results have been reported regarding the roles of netrin-1 and netrin-4 in angiogensis in previous studies. On one hand, some studies report the anti-angiogenic effects of netrin-4 on VEGF-stimulated endothelial cells [9], human microvascular endothelial and human pancreatic carcinoma cells via the inhibition of Akt and JNK1/2 phosphorylation [10]. Moreover, netrin-4 is anti-angiogenic and inhibits endothelial cell function by binding to neogenin and recruiting Unc5B [9]. Additionally, netrin-4 can also cause the *in vivo* filopodial retraction of the endothelial cell [12]. Netrin-1 has also been shown to inhibit postnatal angiogenic sprouting and neovascularization by activating the Unc5B receptor [13]. In addition, netrin-1 or netrin-4 overexpression in tumor cells delays tumor angiogenesis in various animal models [9, 13, 14]. On the other hand, studies also demonstrated that netrin-4 acts as a pro-angiogenic factor [8, 15–17]. It has been reported that netrins stimulate cell proliferation and migration in primary endothelial cell (EC) cultures [11, 17] and vascular smooth muscle cells (VSMCs) [18]. These pro-angiogenic effects are independent from cognate netrin receptor expression in EC [11, 17], and neogenin is the only receptor involved in netrin signaling in VSMCs [18]. The same group also reported that netrin-4 induces lymphangiogenesis [16]. In addition, netrin-4 has been shown to enhance angiogenesis after cerebral ischemia [8]. As a result the exact role of netrin-4 in angiogenesis remains uncertain. Netrin-4 has been detected in mouse cornea, and the deletion of netrin-4 in Ntn4 homozygous null (Ntn4−/−) mice did not disrupt Bowman’s membrane (BM) in the cornea [19], suggesting that netrin-4 is not a structural element of ocular BM. Instead, netrin-4 might function in regulating epithelial cell proliferation or migration.

In this study, we first used human umbilical vein endothelial cells (HUVECs) as a model of vascular endothelial cells to assess the effects of netrin-4 *in vitro*. Next, we used a well-established corneal neovascularization model—the alkali-burn model—to assess the therapeutic effects of netrin-4 on corneal angiogenesis. Our results suggest the crucial role of netrin-4 in the regulation of corneal angiogenesis.

## Materials and Methods

### Cell culture

HUVECs were purchased from Promo Cell (Heidelberg, Germany) and cultured in endothelial cell basal medium 2 (EBM2) containing low serum (2% FCS) and endothelial cell growth supplement (Promo Cell). Endothelial cells were used after 2–6 passages. Because the complete medium contains a large amount of netrin-4, and because endothelial cells produce and secrete high levels of netrin-4 in normal culture media [15], the endothelial cells were serum-deprived for 16 hours before the *in vitro* experiments and the effects of netrin-4 were analyzed in the absence of serum. Plates were observed under an inverted microscope to determine confluence and morphology.

### Cell counting kit-8 (CCK-8) assay

The respective HUVEC cell suspensions (100 μL containing 5×10^3^ cells/well) were dispensed in triplicate into 96-well plates and incubated for 24 hours. Netrin-4 was added to the medium at a concentration of 5000 ng/mL. After 72 hours, 10 μ L Cell CCK-8 solution (Dojindo, Kumamoto, Japan) was added to the wells, and the plates were incubated for 1 hour. Absorbance (i.e., optical density [OD]) was read at 450 nm using a universal microplate reader (Bio-Tek, Winooski, VT, USA), and a graph of OD at 450 nm against concentration was plotted. Each mark represents the mean of the collected readings, and the procedure was repeated at least three times. Within 4 hours, OD at 570 nm was determined using a microplate reader (ELX800, BIO-TEK Corporation, USA).

### In vitro tube formation assay

HUVECs were serum-starved in EBM2 medium (0.1% FBS without growth factor) (Lonza, USA) for 12 hours, and HUVECs were seeded to a density of 10,000 cells/well on growth factor-depleted Matrigel (BD Biosciences, NSW, Australia) in 24-well plates. Netrin-4 (100, 500, 1000, or 5000 ng/mL) or the PBS (control supernatant) was added, and the results were quantified 6 hours later. Microscopic fields containing the tube structures that formed in the gel were photographed at low magnification (10×). At least five fields in each well were examined. Before they were photographed, cells were fixed with 4% paraformaldehyde. Tube length was quantified using Image J software.

### Flow cytometric cell-cycle analysis

HUVECs were grown under the above-described conditions in 35-mm dishes until confluent. Netrin-4 was added to the medium at a concentration of 100, 500, 1000, or 5000 ng/mL. Incubation was continued for 72 hours at 37°C. Cells were removed using 0.05% trypsin in PBS buffer, pelleted (centrifugation at 300 *g* for 5 minutes), washed twice in 1% BSA in PBS, resuspended in 1% BSA in PBS, and fixed in 70% cold ethanol. A flow cytometer (FACScan; BD Biosciences, Franklin Lakes, NJ, USA) was used to acquire all data.

### Cell apoptosis analysis

Duplicate HUVECs were plated onto 60-mm culture dishes at a density of 2×10^5^. When the cells achieved 70–80% confluence, HUVECs were treated with 100 ng/mL, 500 ng/mL, 1000 ng/mL, or 5000 ng/mL netrin-4. Incubation was continued for 72 hours at 37°C. Apoptosis was analyzed using an Apoptosis and Necrosis Assay Kit with Hoechst 33342 and PI staining (Beyotime Institute of Biotechnology, Shanghai, China). Briefly, the cultured HUVECs were trypsinized, washed twice with PBS, and then suspended in 100 μL cell-staining buffer. Then, 5 μL Hoechst 33342 and 5 μL of 10 μg/mL PI was added, and the cells were incubated at 4°C or the cells were kept on ice for 20 minutes in the dark. After incubation, apoptotic cells were immediately analyzed using flow cytometry.

### Wound closure assay for assessing migration

HUVEC migration was assessed using the wound-healing assay as previously described [20, 21]. HUVECs were seeded (1×10^5^ cells/well) in duplicate to 1% gelatin-coated 24-well plates (Corning, Schiphol, Netherlands). Cells were grown until confluence, and a scratch wound was applied in two perpendicular directions using a sterile pipet tip (200-μL yellow tip), thereby creating linear, cross-stripe scrapes that were 2 mm apart. Monolayers were washed with PBS to remove floating cells, the experimental medium (containing 100, 500, 1000, or 5000 ng/mL netrin-4) was added, and the cells were incubated for an additional 24 hours. Cell migration to where the scrapes were introduced was photographed at different time points using an inverted microscope.

### Cell invasion assay

HUVEC invasion across Matrigel was evaluated in the invasion chambers. Cell inserts (8-mm pore size, 6.5-mm diameter; Corning) were coated with 15–25 mL Matrigel and placed in 24-well plates. HUVECs were plated at a density of 2×10^5^ cells/well in the upper chamber, while either 0 or 800 μL netrin-4 (100, 500, 1000, or 5000 ng/mL) was added to the lower chamber and incubated for 24 hours. After washing with PBS, the non-invaded cells in the inserts and Matrigel were removed from the upper surface of the filter by wiping with a cotton bud. Inserts were then fixed in 4% formalin for 10 minutes at room temperature and stained with 4′, 6-diamidino-2-phenylindole (DAPI). Cells were observed under a fluorescent microscope. Cells that migrated to the lower surfaces were counted at 40× magnification in five predetermined fields.

### Alkali-burned rat corneas and treatment

Wistar rats (180–220 g; 2 months old; male; n = 30) were purchased from Shanghai Shilaike Laboratory Animal Co, Ltd., Shanghai, China. Animal experiments were carefully performed in accordance with the guidelines of the Association for Research in Vision and Ophthalmology (ARVO) Statement for the Use of Animals in Ophthalmic and Vision Research, and all animal experimental procedures were approved by the Experimental Animal Committee of Xiamen University (approval ID: XMUMC2013-02-1). Animals were housed in a temperature, humidity, and light controlled room. Food and water were available ad libitum. All rats were confirmed to be free of ocular diseases before experimentation. Alkali burns were applied to the rat corneas as previously reported [7, 22, 23]. The procedures of corneal alkali burn were briefly as follows: The rats were anesthetized with an intraperitoneal injection of 40 mg/kg pentobarbital and received topically administration with a drop of tetracaine. A round filter paper (3.5 mm in diameter) soaked with 1 N NaOH was placed on the center of the corneal surface for 30 sec to induce alkali burn. The ocular surface was then rinsed with 10 mL PBS.

After applying the alkali burn injury, animals were randomly divided into four equally sized groups (7 rats per group). Rats in two groups received the topical administration of PBS (10 μ L was administered four times per day, for 14 or 24 consecutive days), and the rats in the other two groups received the topical administration of recombinant mouse netrin-4 (R&D Systems, Minneapolis, MN, USA) using a pipette (10 μ L was administered four times per day at a concentration of 5000 ng/mL in PBS, for 14 or 24 consecutive days). Treatments were administered for 14 consecutive days, all eyes were observed on day 1, 4, 7 and 14 with slit lamp microscopy as described below for evaluation of corneal NV and inflammation and measurement of corneal epithelium damage. The rats were sacrificed with an overdose of pentobarbital sodium on postoperative day14, eye balls were removed, and the corneas were dissected and stored at -80°C until histological examination or protein extraction. In the fourth group, treatment started at day 10 after injury and were continued for another 14 days in order to observe the regressive effects of netrin-4 on corneal neovascularization. All eyes were observed on day 10, 14, 17, 20 and 24 with slit lamp microscopy as described below for evaluation of corneal NV and inflammation. The rats were sacrificed with an overdose of pentobarbital sodium on postoperative day 24, eye balls were removed, and the corneas were dissected and stored at -80°C until histological examination or protein extraction.

For the histology examination, the corneas were embedded in OCT, cross-sectioned, Hematoxylin and Eosin staining (H&E staining) was conducted as described below. For the Western blot analysis, the whole cornea tissue including limbal (about 0.5 mm width) area was carefully dissected and Western blotting was conducted as described below.

### Slit-lamp microscopic observation

Animals were examined daily using a slit-lamp microscope after the alkali burns were applied. Corneal images were obtained by an experienced researcher. Corneal epithelial defects were determined by staining the ocular surface with 0.1% fluorescein sodium and observation under cobalt blue light. Images were processed using image-processing software (Image Pro Plus V6.0; Media Cybernetics, Silver Spring, MD, USA). Corneal neovascularization was quantified by calculating the wedge-shaped area (S) of the vessel growth using the following formula:

$$\text{S }=\text{ C}/12\times 3.1416\times \left[{\text{r}}^{2}-\;{\left(\text{r}-\text{ I}\right)}^{2}\right]$$

where S is the area, C is time, I is the radius to the border of the vessel, and r is the radius of the cornea [24]. The inflammatory index was analyzed according to various parameters, including ciliary hyperemia, central corneal edema, and peripheral corneal edema, as previously described [25].

### Rat cornea samples and HUVEC cell culture supernatant collection and Netrin-4 ELISA

The entire corneal tissue was carefully dissected and extracted with cold PBS (PH7.4), homogenized by grinder, centrifugation 20min at the speed of 3000 r.p.m., remove supernatant. HUVEC cell culture supernatant detects secretory component, collect sue a sterile container, centrifugation 20min at the speed of 3000 r.p.m. We analyzed the rat cornea and HUVEC supernatant netrin-4 levels using a rat netrin-4 ELISA kit (Hui Ying Biotechnology Co., Ltd., Shanghai, China) according to the manufacturer’s protocol.

### Histology Analysis

Rat cornea samples were fixed overnight in 4% paraformaldehyde (PFA) in PBS, followed by dehydration in a series of ascending alcohol and paraffin embedding. Deparaffinized sections (5μm) were stained with hematoxylin and eosin (H&E).

### Apoptosis Detection Assay

To detect apoptosis of the corneal cells, the corneas were embedded in OCT, cross-sectioned, and subjected to terminal deoxynucleotidyl transferase-mediated nick end labeling (TUNEL) staining (Dead End Fluorometric TUNEL system; Promega, Shanghai, China), according to the manufacturer’s protocol. Cellular nuclei were stained with 4-6-Diamidino-2-phenylindole (DAPI), and apoptotic cells were examined under a laser confocal microscope (Fluoview 1000, Olympus, Tokyo, Japan). The cellular nuclei and apoptotic cells were counted in three sections from each sample.

### Western blot assay

To determine protein expression levels in the cornea, the entire corneal tissue was carefully dissected and extracted using cold RIPA buffer and proteinase inhibitor cocktail (Merck, Darmstadt, Germany). Equal amounts of proteins were extracted from the lysates and subjected to electrophoresis on 10% sodium dodecyl sulfate-polyacrylamide gels, then electrophoretically transferred to polyvinyl difluoride (PVDF) membrane. After 30 minutes of blocking in 2% BSA, the blots were incubated with primary antibodies against netrin-4 (1:200; Santa Cruz, USA), vascular epidermal growth factor (VEGF; 1:200; Santa Cruz, USA), PEDF (1:200; Santa Cruz, USA), and NF-KB p65 (1:200; Santa Cruz, USA). β-actin (1:10,000; Bio-Rad, Hercules, CA, USA) was used as the loading control. After three washes with Tris-buffered saline with 0.05% Tween 20 for 10 minutes each, the membranes were incubated with horseradish peroxidase (HRP)-conjugated secondary antibodies and Ig G (Bio-Rad) and rabbit anti-goat antibodies (1:5000; Dako, Shanghai, China) for 1 hour at room temperature. The results were visualized using enhanced chemiluminescence reagents and recorded on film.

### Statistical Analysis

Summary data are reported as the mean ± SD. The inflammatory index and the area and length of the corneal neovascularization were analyzed using ANOVA followed by Bonferroni post hoc comparison. Group means were analyzed using the student *t* test, and *p* < 0.05 is considered statistically significant. Statistical analyses were conducted using GraphPad Prism for Windows (version 5.00; GraphPad Software Inc., USA).

## Results

### Netrin-4 concentrations in rat cornea and HUVEC

**Fig 1:**
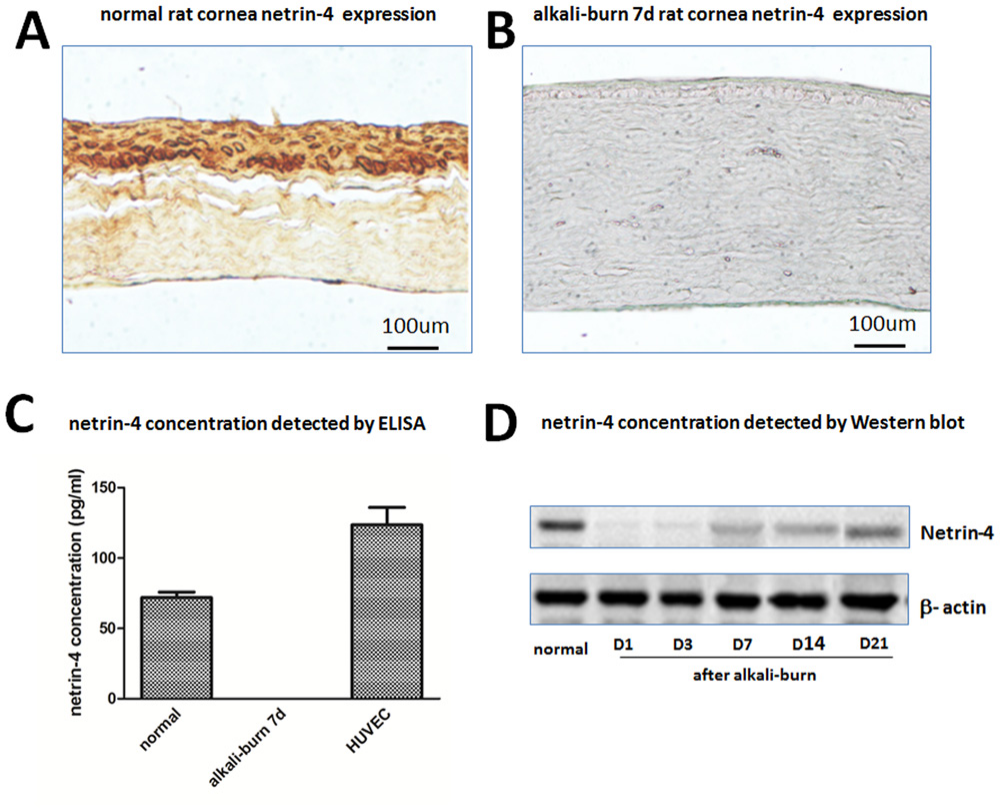
Levels of netrin-4 in endothelial cells, and in cornea before and after alkali-burn were detected by ELISA, IHC and Western blot. Netrin-4 expression in normal (A) and after alkali-burn rat cornea (B) by immunohistochemical staining. Netrin-4 concentrations in rat cornea and HUVEC (C). Netrin-4 concentration in normal rat cornea and different time after alkali-burn treatment.

The levels of netrin-4 in endothelial cells and cornea before and after alkali-burn were first determined by using ELISA, IHC and Western blot. The level of netrin-4 in HUVEC is about 123pg/mL (Fig 1C), and it is about 72pg/mL in normal rat cornea (Fig 1C), which could not be detected 7 days after alkali-burn (Fig 1C). Meanwhile, we performed immunohistochemical staining and found that netrin-4 (Fig 1A) was expressed in both normal corneal epithelium and stroma. Strong staining was detected in the membrane of epithelial cells. But there wasn’t netrin-4 expression 7 days after alkali-burn (Fig 1B). Western blot results showed that netrin-4 expressed in normal rat cornea, after alkali-burn treatment there was no expression on day 1and 3. After 7days, there was weak netrin-4 expression in rat cornea. Then netrin-4 expression increased slowly at day 14 and 21(Fig 1D).

### Netrin-4 decreases HUVECs viability in a dose-dependent manner by regulating HUVEC proliferation and apoptosis

**Fig 2:**
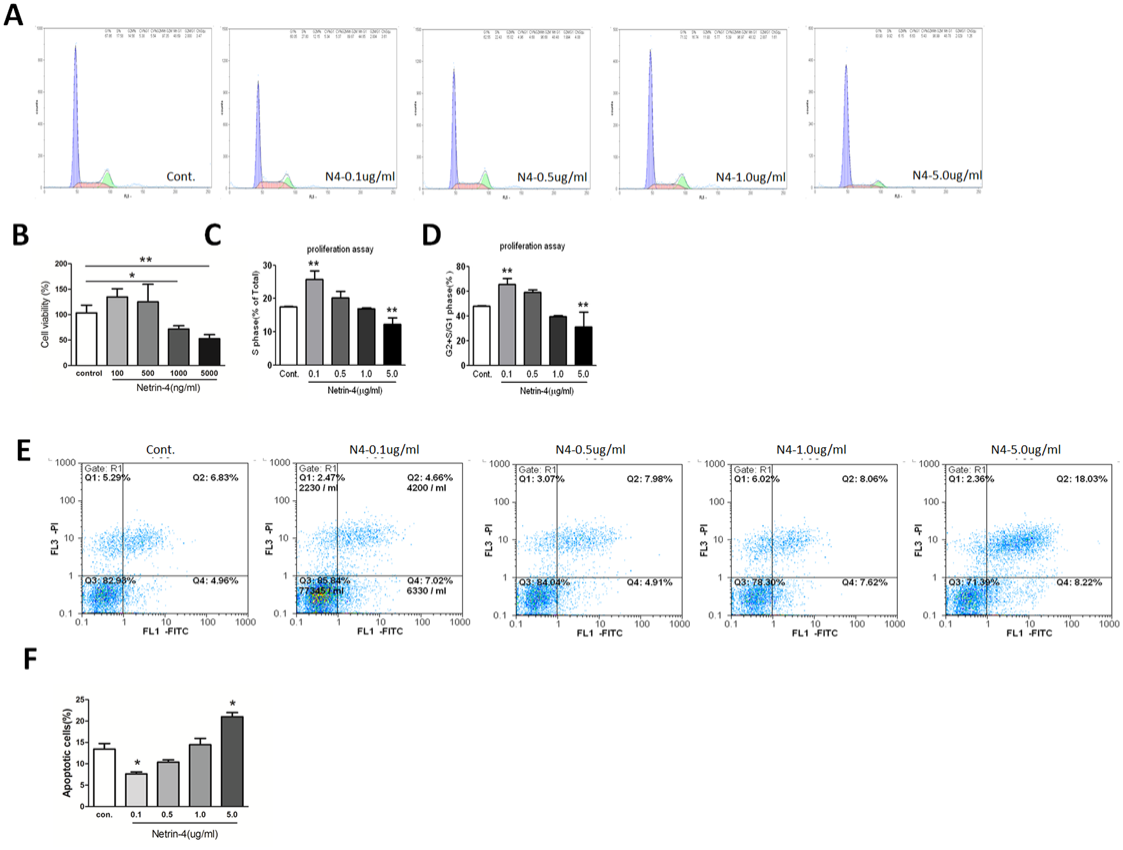
Cell viability, HUVEC proliferation and apoptosis on different doses of netrin-4 was detected by using the CCK-8 assay and flow cytometry. (A) Cultured HUVECs were serum-starved for 24 h followed by 72 h culture in serum free media with or without dosage netrin-4 protein. The cells were then harvested, stained with propidium iodide (PI), and analyzed by flow cytometry. The cell proliferation rate was expressed as the percentage of cells in the S phase and as the G2 + S/G1 ratio (** *p* < 0.01). (B) Cell viability detected by CCK-8. (C) S phase percentage of cells. (D) G2 + S/G1 ratio. (E) Apoptosis assay by flow cytometry, analysis with annexin V-FITC / PI double staining. The numbers in the upper left, upper right, lower left and lower right quadrants represent the percentage of necrotic cells (annexin V positive, PI positive), advanced apoptotic cells (annexin V negative, PI positive), viable cells (annexin V negative, PI negative) and early apoptotic cells (annexin V positive, PI negative), respectively. The values represent the mean percentages of apoptotic cells in different groups (* *p* < 0.05). (F) Quantitative results of the apoptotic cells. Each value represents the mean ± SD, n = 3. * *p* < 0.05; ** *p* < 0.01; compared with the control group.

We first measured the cell viability with CCK-8 assays in the presence of different concentrations of netrin-4 (100, 500, 1000, 5000 ng/mL), which reduced HUVEC viability at 1000 and 5000 ng/mL compared with the control (Fig 2B). The effects of netrin-4 on cell cycle were assessed using flow cytometry. After treating for 72 hours, cells treated with 100, 500, 1000, and 5000 ng/mL netrin-4, 5000ng/mL netrin-4 decreases the percentage of cells in S phase as well as the ratio of cells in G2+S phase compared with G1 phase. However, the opposite effects were observed at the concentration of 100 ng/mL (Fig 2A, 2C and 2D). In addtion, HUVECs were starved overnight and then treated with 100, 500, 1000, and 5000 ng/mL netrin-4, and apoptosis was analyzed using flow cytometry. Representative dot plots of annexin V/PI staining are shown (Fig 2E). The lower left quadrant shows the vital population, the lower right quadrant shows the apoptotic population (annexin V+/PI-), and the upper right quadrant shows the late apoptotic/necrotic population (annexin V+/PI+). The total number of apoptotic HUVECs increased after treatment with 5000 ng/mL netrin-4, and accordingly the total percentage of apoptotic cells increased from 12.12 ± 0.27% to 20.45 ± 0.51% at the high dosage (Fig 2E). The results demonstrate that netrin-4 promotes HUVECs apoptosis at the concentration of 5000 ng/mL but decreases HUVEC apoptosis (Fig 2F).

### Netrin-4 inhibited HUVECs tube formation

**Fig 3:**
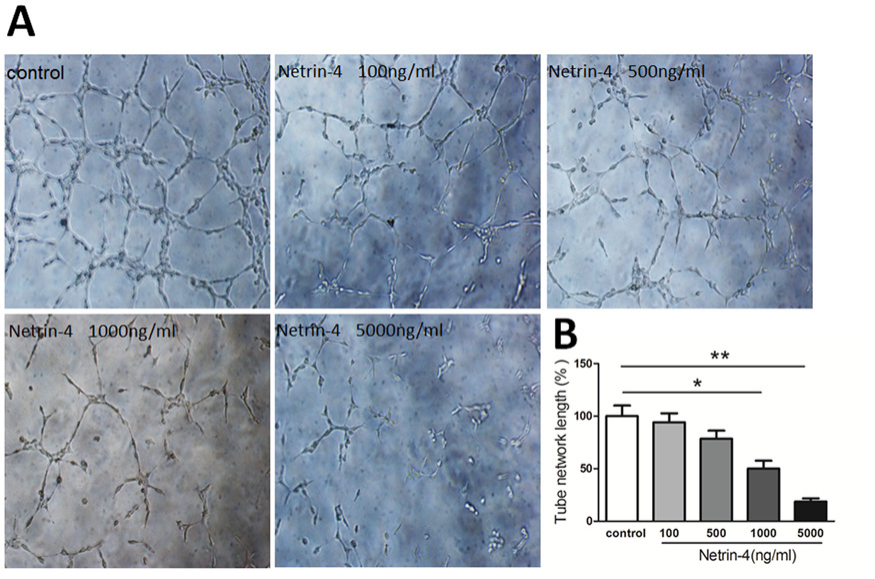
Effect of netrin-4 on HUVECs tube formation. Tube formation was assessed in tubulogenesis assay in vitro model, HUVECs were serum-starved with EBM2 medium (0.1% FBS, no growth factor) (Lonza) for 12 hours. HUVECs (1.5×10^4^) were then cultured in a 24-well plate (Gibco) coated with 150 mL Matrigel Basement Membrane Matrix GFR (BD Biosciences). Different concentrations of netrin-4 (100, 500, 1000, and 5000 ng/mL) or PBS (control) were added, and the results were quantified 6 h later using Image J. (B) Representative images out of three independent experiments are shown. * *p* < 0.05; ** *p* < 0.01. Each experiment was performed three times and representative pictures are shown. Data are expressed as mean ± SD.

*In vitro* angiogenesis assay was performed according to a previously published protocol [26]. We first assessed the effects of different concentrations of netrin-4 (100, 500, 1000, and 5000 ng/mL) on HUVEC tube formation. The results show that 5000 ng/mL netrin-4 significantly inhibited HUVEC tube formation (Fig 3A and 3B). Therefore, 5000 ng/mL netrin-4 was used in the following experiments.

### Netrin-4 effects on HUVEC migration and invasion

**Fig 4:**
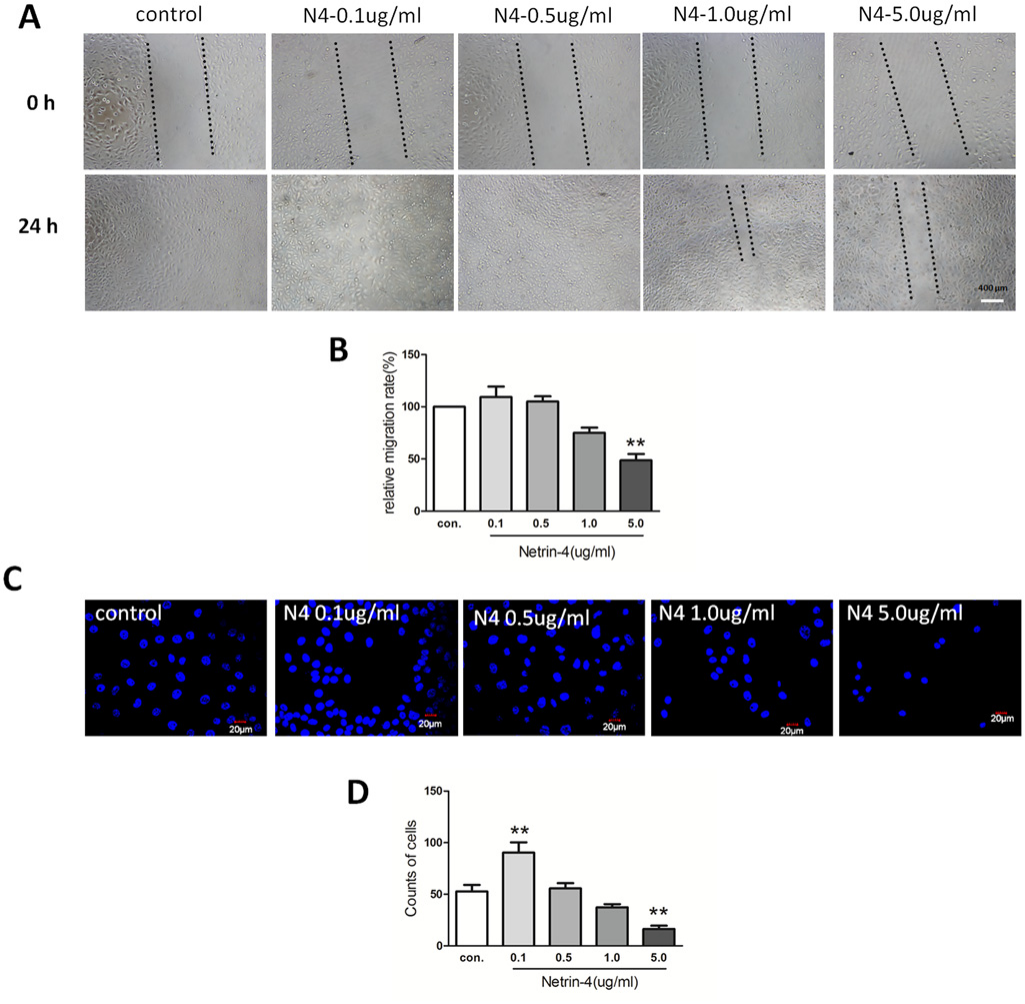
Effect of netrin-4 on HUVECs migration and invasion. HUVEC were treated during 24 h in medium without serum, in the presence of EBM2 alone or EBM2 with different concentrations of netrin-4. (A)Pictures were taken at 0 h and 24 h after insert removing. (B) Results are expressed as percentage of wound closure (percentage closure) (mean ± SD). (C) Netrin-4 affects HUVEC migration in a transwell assay. HUVEC cells were allowed to migrate for 24 h at 37°C in a 5% CO_2_ humidified incubator. After 24 h, the cells that migrated across the membrane were stained with DAPI and the fluorescence intensity was measured. (D)The % invasion is compared to the fluorescence intensity of migrated cells in the absence of netrin-4 (** *p* < 0.01). Each experiment was performed three times and a representative picture of each condition is shown. Data are expressed as mean ± SD.

After verifying the effects of netrin-4 on cell proliferation, we assessed its effects on HUVEC migration using a scratch-wound model. After the scratch wound was created, the wounds were allowed to heal with or without netrin-4. The control wounds closed within approximately 24 hours, while the group treated with 5000 ng/mL netrin-4 did not heal within 24 hours. There was still 980 μm blank in the netrin-4-treated group at 24 h. After 24 hours treatment, the migration rates were 42.6 and 26.1 μm/hour in the control and netrin-4–treated groups, respectively (Fig 4A and 4B). Based on the results of the transwell invasion assays, the 5000 ng/mL netrin-4–treated group demonstrated lower invasive abilities than the controls, but 100ng/mL netrin-4 increases HUVEC invasive abilities (Fig 4C and 4D). These results suggest that netrin-4 exhibits a dose-dependent effects on HUVEC proliferation, migration, apoptosis, and invasion. At 5000ng/mL, netrin-4 can inhibit HUVEC proliferation, migration, invasion, but increase apoptosis. However, at lower dosage 100ng/mL, the role of netrin-4 is opposite.

### Netrin-4 prevents corneal neovascularization in alkali-burned rat corneas

**Fig 5:**
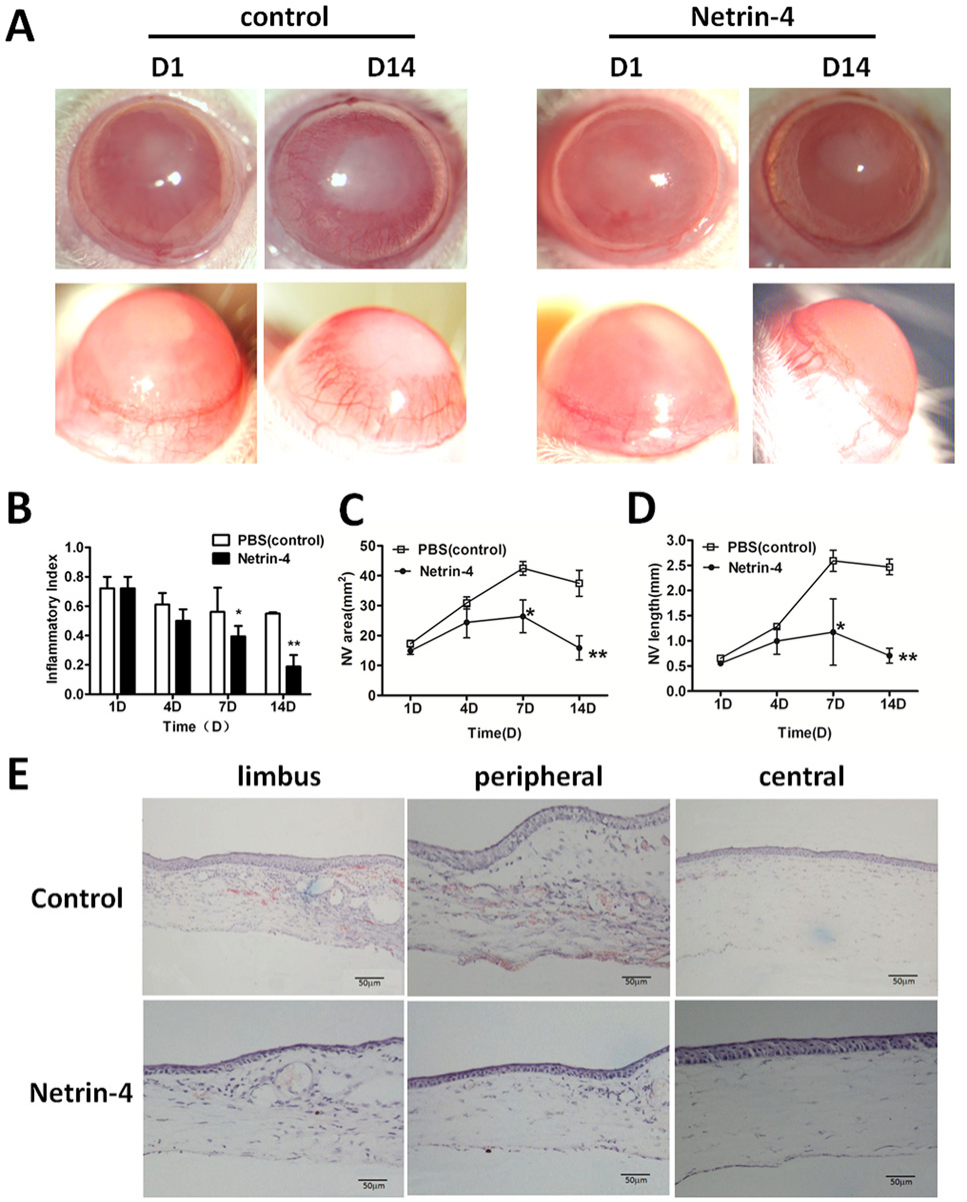
Netrin-4 prevents corneal neovascularization after alkali burns. (A) One day after the alkali burns, the central corneal stroma of the rats appeared opaque and edematous in both groups. On day 14 after the injury, new blood vessels reached the central corneas in the control group, and there was a remarkable decrease in corneal transparency. In contrast, there was only slight new blood vessel formation in the limbal areas in the netrin-4 treatment group, and the corneas remained transparent on day 14. (B) The inflammatory index of the ocular surface declined from day 1 to day 14 in both groups. However, it was significantly lower in eyes treated with netrin-4 on day 7 and day 14 (* *p* < 0.05 and ** *p* < 0.01). (C) New blood vessel formation area (NV area) in the control group increased from day 1 to day 7, and there was a mild decrease on day 14 after the alkali burns. In contrast, corneas treated with netrin-4 showed only a mild increase in NV area on day 7. There was a significant difference between the two groups on day 7 and day 14 (** *p* < 0.01). (D) The average new blood vessel length (NV length) increased from day 1 to day 7 and decreased on day 14 in the PBS group, while the NV length continued to be very short in the netrin-4 treatment group. There were significant differences between the two groups on day 7 and day 14 (** *p* < 0.01). (E) H&E staining showed prominent new blood vessel formation from the limbal areas to the central corneas in the control group on day 14, which was well-indicated by the red blood cells that remained in the blood vessels. However, the netrin-4 treatment group only showed a few blood vessels in the limbal areas and none in the peripheral and central corneas.

Alkali burn has been shown to cause severe neovascularization in the cornea [27]. We evaluated the effect of netrin-4 on corneal neovascularization, and we used the alkali burn model to compare vessel formation between injured corneas that received netrin-4 treatment and controls. The onset of peripheral neovascularization occurred on day 1 after the alkali burns were applied to both groups (Fig 5A). The inflammatory index showed slight decrease from day 1 to day 14 in PBS group, while there was dramatic reduction in the netrin-4 group, and there was significant difference between the two groups at day 7 and day14 (Fig 5B).

In the control group, there was an ingrowth of new blood vessels toward the peripheral corneas on day 4, which reached the central corneas on day 7, and new blood vessels were detected on day 14 (Fig 5C and 5D). In contrast, corneas treated with netrin-4 demonstrated only a mild increase in new blood vessels, which was maintained at low levels throughout the study period (Fig 5A). The new blood vessel areas and lengths were much lower than control corneas on days 7 and 14 (Fig 5C and 5D). Hematoxylin-eosin (H&E) staining showed persistent, new blood vessel formation in the anterior part of the corneal stroma in the control group on day 14 after applying the alkali burns, which was clearly indicated by the remaining red blood cells in the blood vessels. However, the netrin-4–treated group developed only a few blood vessels in the limbus and none in the peripheral or central corneas (Fig 5E).

### Netrin-4 promotes the regression of corneal neovascularization and inhibits apoptosis in alkali-burned rat corneas

**Fig 6:**
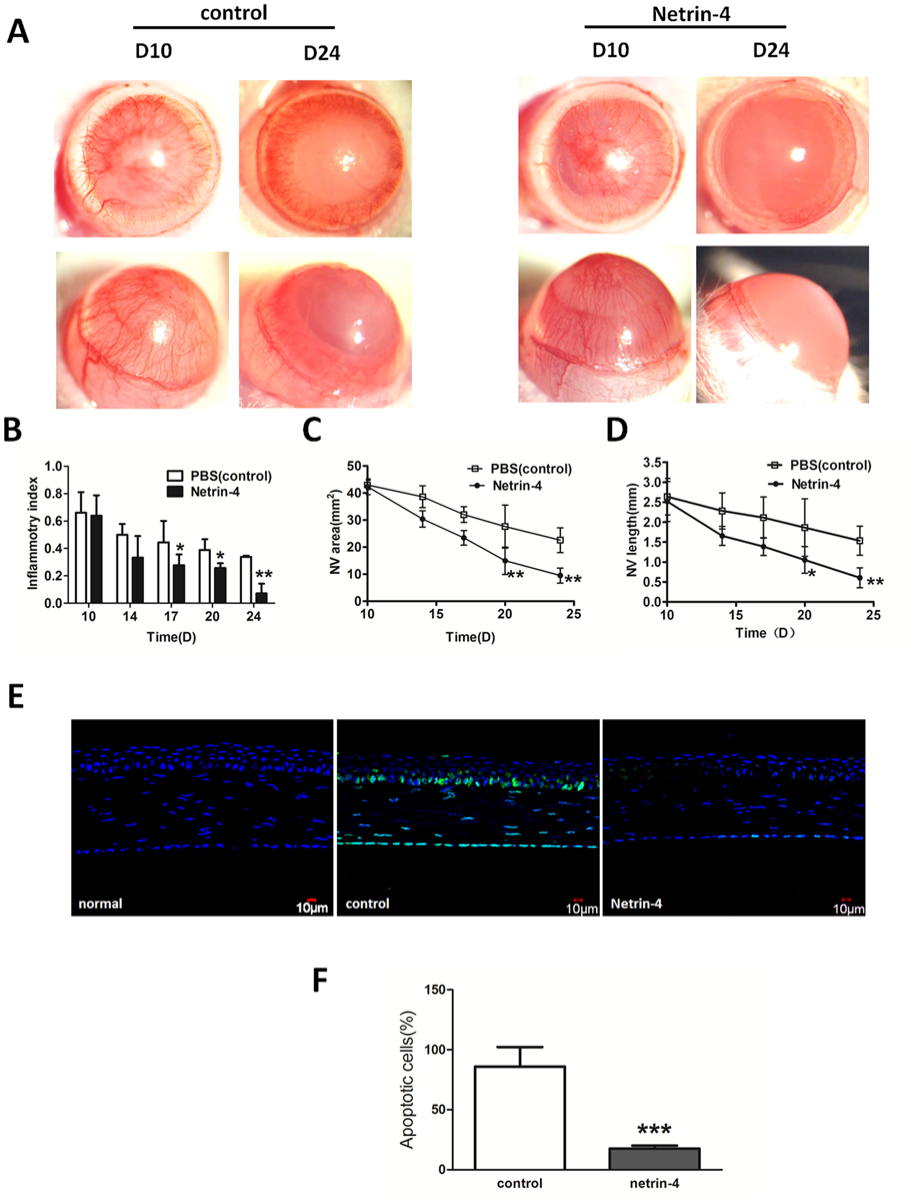
Netrin-4 promotes the regression of corneal neovascularization and inhibit apoptosis after alkali burns. (A) Ten days after the injury, dense neovascularization reached the central cornea. In this experiment, netrin-4 treatment began on day 10. By day 24, the new blood vessels regressed from the central cornea to the peripheral cornea in the control group. In contrast, almost all the new blood vessels had regressed to the limbal area in the netrin-4 treatment group by day 24. (B) The inflammatory index continuously decreased from day 10 to day 24 in both groups, while the index was significantly lower in the netrin-4 treatment group on days 17, 20, and 24 (* *p* < 0.05). (C) The NV area gradually reduced from day 10 to day 24 in both groups. There was a dramatic decrease of NV area in the netrin-4 treatment group on day 20 and day 24, and there was a significant difference between the two groups (* *p* < 0.05 and ** *p* < 0.01). (D) The average NV length was continuously reduced from day 10 to day 24 in both groups, and the length was shorter in the netrin-4 treatment group on days 17, 20, and 24 than in the other group (* *p* < 0.05 and ** *p* < 0.01). (E) Netrin-4 reduced alkali burn-induced apoptosis of corneal cells. (F) A statistical analysis of the apoptotic cells on day 7 between the two groups showed significant difference (*** *p* < 0.001).

To determine if netrin-4 induces the regression of well-formed corneal neovascularization, netrin-4 treatment was started on day 10 after applying alkali burns. In the rat alkali-burn model, corneal neovascularization peaked 10 days after injury (Fig 6C). Thereafter, gradual blood vessel regression was noted in the control group (Fig 6C). By 24 days post injury, the blood vessels regressed from the central cornea to the peripheral cornea in the control group. On the other hand, the rapid regression of corneal neovascularization was noticed when the eyes were treated with netrin-4, and almost all of the blood vessels regressed to the limbal area 24 days after treatment (Fig 6A). The inflammatory index showed gradual decrease in the PBS group, while it was further decreased in the netrin-4 group at days 17, 20, and 24 (Fig 3B). The area of corneal neovascularization (Fig 6C) and length (Fig 6D) on day 24 were significantly lower in the netrin-4 group in comparison with the control group. Meanwhile, to determine the effect of netrin-4 on alkali-burn induced apoptosis, we performed TUNEL assay on corneas treated with PBS or netrin-4 post alkali injury. As expected, there were no apoptotic cells present in normal rat corneas (Fig 6E). After alkali-burn, there were still many apoptotic cells in the basal epithelia, stromal, and endothelia at day 7. However, when the corneas were treated with netrin-4, there were much fewer apoptotic cells compared with the PBS control, and the majority of the apoptotic cells resided in the endothelia (Fig 6E). Statistical analysis showed a significant difference of apoptotic cells between the two groups (Fig 6F)

### Netrin-4 affects the expression of VEGF, PEDF, NF-KB p65, PMN and ED1 (CD-68) in alkali-burned rat corneas

**Fig 7:**
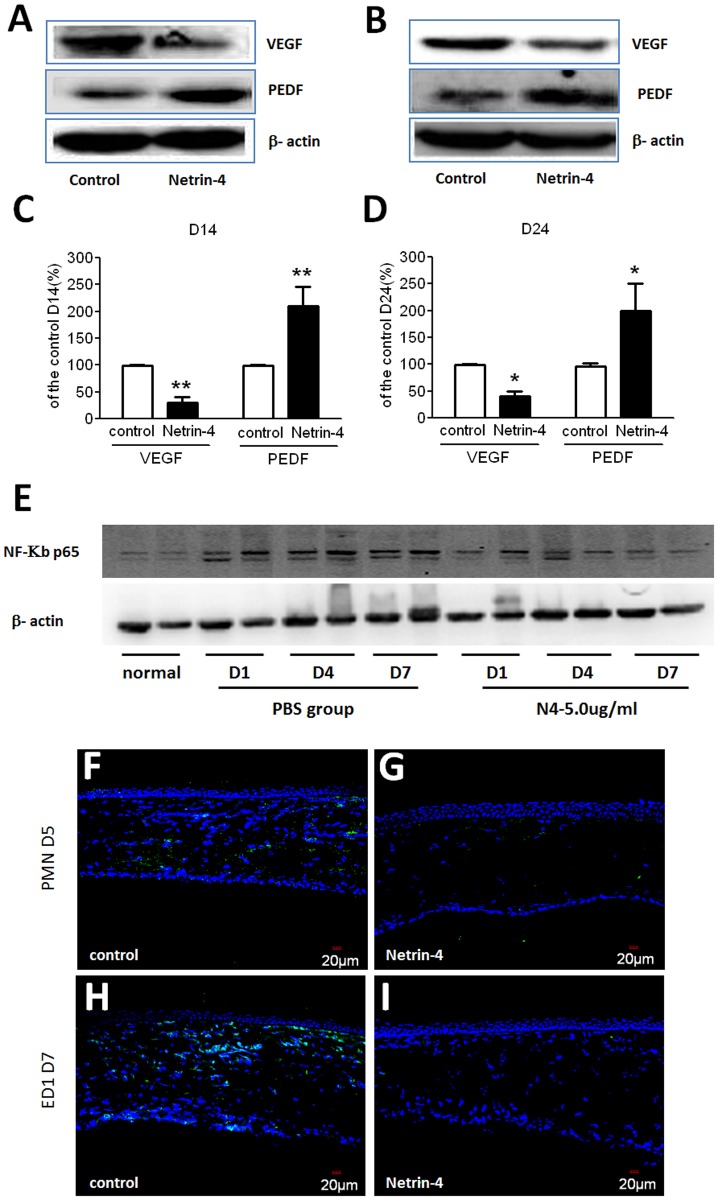
Effect of netrin-4 on VEGF, PEDF, NF-KB p65, PMN and ED1(CD68) expression after corneal alkali burns. (A) Western blot analysis results showed that VEGF was expressed at low levels in normal rat cornea, while there was a dramatic increase at day 14 (D14) after alkali burns in the control group. In the late stage treatment experiment, there was a decrease in VEGF on day 24 (D24). In the netrin-4 treatment group, there was a dramatic down regulation of VEGF on day 14 and day 24. PEDF was expressed in normal corneas and was dramatically decreased on days 14 and 24 after alkali burns, whereas it was restored after netrin-4 treatment. Densitometry of protein expression compared with β-actin showed significant differences between the control group and the netrin-4 treatment group in (B) VEGF and (C) PEDF on days 14 and 24 (** *p* < 0.01). (E) NK-KB p65 expression detected by western blot. (F) PMN and ED1(CD68) immunostaining after alkali burn day 5, 7.

It is well established that corneal neovascularization is tightly regulated by a dynamic, natural equilibrium between local proangiogenic and antiangiogenic molecules [28–31]. Among them, VEGF and PEDF are considered to play major roles. To investigate the mechanism hownetrin-4 inhibits and reverses corneal neovascularization after alkali burns application, we used Western blot analysis to assess VEGF and PEDF protein levels. Our results show that VEGF is expressed at low levels in normal rat corneas, but there was a dramatic increase in the control group on day 14 after alkali burns. In the later stage, there was a decrease in VEGF expression on day 24. After netrin-4 treatment, VEGF expression was lower than that of the control group on days 14 and 24 (Fig 7A and 7B). PEDF was expressed in the normal corneas and dramatically decreased on days 14 and 24 after alkali burns. However, PEDF was restored in the netrin-4 treatment group, although its expression was still lower than that observed in the normal corneas (Fig 7A and 7D). To further illustrate the function of netrin-4 in corneal inflammation, we conducted western blot NF-KB p65 and immunostaining of PMN and ED1(CD-68) antibodies to detect inflammation, neutrophil and macrophage infiltration after alkali burns. In the normal rat cornea, NK-KB p65 expressed lower than after alkali-burn. However, netrin-4 significantly decreased NK-KB p65 expression at day 1, 4, 7 (Fig 7E). Meanwhile, we detected netrin-4 inhibits neutrophil and macrophage infiltration by immunostaining (Fig 7F).

## Discussion

Netrins have been shown to play various important roles in central biological processes including cell guidance, adhesion, differentiation, and survival as well as in angiogenesis. Netrin-1 and netrin-4 are the most extensively studied members of the netrin family [32]. We previously reported that netrin-1 acts as an anti-angiogenic factor by inhibiting alkali-burn–induced cornea neovascularization in rats [7]. In this study, we investigated the effects of netrin-4, which is a member of the same protein family, on *in vitro* and *in vivo* cornea neovascularization in rats. The level of netrin-4 in normal rat cornea is about 72 pg/ml. Meanwhile, I also detected the concentration of netrin-4 7days after alkali-burn, which cannot be detected. We applied 5000 ng/mL netrin-4 seems quite far from physiological concentrations. But after the tear dilution and incomplete absorption, we detected the concentration of netrin-4 at14 days after alkali-burn and netrin-4 treatment is about 64 pg/ml, and 24 days is about 72 pg/ml. They almost recovered to the normal level. We, and others, have analyzed the *in vitro* effects of netrin-4 on cells [9, 18] and exogenous netrin-4 [16, 19]. However, this is the first study to evaluate the effects of netrin-4 on cornea neovascularization. Netrin-4 has been detected in mouse cornea, and the deletion of netrin-4 in Ntn4 homozygous null (Ntn4−/−) mice did not disrupt Bowman’s membrane (BM) in the cornea[19], suggesting that netrin-4 is not a structural element of ocular BM. Instead, netrin-4 might function in other ways by regulating epithelial cell proliferation or migration.

Netrin-4 is richly deposited in vascular BM. Vascular endothelial cells are most likely the cellular source of netrin-4, as vascular endothelial cells are reported as a source of netrin-4 (according to *in situ* hybridization [33] and microarray analyses [34]). We also determined how netrin-4 affects HUVECs, and we investigated the effects of netrin-4 on tube formation, proliferation, apoptosis, migration, and invasion in HUVECs. The wound-healing process that takes place in cellular monolayers involves complex processes, including migration, and proliferation. Different doses of netrin-4 alter tube formation and the rates of adhesion and/or migration of HUVECs. In our study, we found that 0.1 μg/mL netrin-4 promotes HUVEC survival, proliferation, migration and invasion, but inhibits apoptosis and demonstrates pro-angiogenic effects *in vitro*. These findings are consistent with recent studies illustrating that netrin-4 stimulates the migration of both human microvascular endothelial cells (HMVECs) and HUVECs in a dose-dependent manner *in vitro* [17] and netrin-4 induces the *in vitro* proliferation, migration, adhesion, tube formation, and survival of human lymphatic dermal human microvascular endothelial cells (HMVEC-dLys) at physiological doses (i.e., 500 ng/mL) [16]. In addition, 5000 ng/mL netrin-4 inhibited HUVEC survival (Fig 2B), proliferation (Fig 2C), migration (Fig 4A) and invasion (Fig 4C). Netrin-4 also induced apoptosis (Fig 2E) and demonstrated anti-angiogenic *in vitro*, which is consistent with a published report that concluded that netrin-4 (25 μg/mL) inhibits HMVEC proliferation and migration [10]; furthermore, netrin-4 has anti-adhesive properties in endothelial cells previously [17]. Based on these data, we conclude that netrin-4 either acts as a pro- or anti-angiogenic factor *in vitro*, depending on dosage, consistent with netrin-4 regulates glioblastoma cell proliferation and migration in a concentration-dependent manner (0, 50, 200, 3000 ng/mL) [35].

Alkali burns produce substantial angiogenesis in the cornea. We found that 5000 ng/mL netrin-4 reduced the corneal inflammatory index (Figs 5B and 6B) and inhibited (Fig 5A, 5C and 5D) and reversed (Fig 6A, 6C and 6D) neovascularization in alkali-burned corneas. This study shows that the potent anti-angiogenic effects of netrin-4 could be used to treat corneal alkali burns. Netrin-4, when applied at different stages of the alkali burn, not only inhibited (Fig 5) but also reversed (Fig 6) corneal neovascularization. This is the first time that netrin-4 has been shown to attenuate corneal neovascularization in a corneal model of alkali burn; this is also consistent with the findings that netrin-4 significantly reduces pathological angiogenesis in Matrigel (6 μg/mL netrin-4) and laser-induced choroidal neovascularization models (1 μ g/mL netrin-4) [9] and netrin-4 overexpression delays tumor angiogenesis and growth. These netrin-4–involved inhibitory effects are associated with decreased tumor cell proliferation and increased tumor cell apoptosis [9, 14]. Netrin-4 most likely negatively regulates proliferation in the cornea during normal corneal development and maintenance [19].

In the early stages of alkali burns, the majority of the central corneal epithelial cells went into apoptosis. However, netrin-4 significantly reduced the number of apoptotic cells, not only in the epithelia, but also among keratocytes and endothelia cells (Fig 6E). Several studies showed that netrins regulate apoptosis via their dependence receptors [36] and downstream signaling pathways involving v-Akt murine thymoma viral oncogene homologue, extracellular signal-regulated protein kinase and apoptosis signal-regulationg kinase 1[5]. Neogenin and UNC5 dependence receptors induce apoptosis in the absence of netrin ligand and inhibit apoptosis when netrin is bound [37].

As shown before, traumatic disorders can disrupt the balance between angiogenic and anti-angiogenic factors in the cornea and tilt the balance toward angiogenesis [38]. Our results demonstrate that netrin-4 can restore balance between VEGF and PEDF that is disrupted by alkali burns. In our study, VEGF was downregulated after netrin-4 treatment, supporting the notion that netrin-4 may affect corneal neovascularization by inhibiting macrophage infiltration. Interestingly, PEDF expression was restored in netrin-4–treated corneas. PEDF is a potent antiangiogenic factor found in retinoblastoma cells, retinal pigment epithelia, iris, and cornea [39]. PEDF promotes endothelial cell apoptosis and also inhibits endothelial cell migration and tube formation [40]. The effect of netrin-4 on the expression of VEGF and PEDF may be a representation of the mechanisms that underlie the inhibitory effect of netrin-4 on corneal neovascularization. Future studies are needed to explore the mechanisms by which netrin-4 regulates PEDF expression. Macrophages act in concert with neutrophils to phagocytose debris and invading pathogenic microorganisms and are a source of growth factors that promote resolution of inflammation as well as cell migration and proliferation for wound healing. On the other hand, neutrophils release into the injured tissue oxidative, hydrolytic, and pore-forming molecules which can damage host cells; macrophages also secrete abundant inflammatory cytokines, chemokines, and angiogenic factors which contribute to angiogenesis and scar formation of the wounded tissue. Therefore, exaggerated or constant influx and presence of neutrophil and macrophage is detrimental [41, 42]. In our study, neutrophil and macrophage infiltration was significantly reduced approximately 1 week post injury, which may have a major impact on the resolution of corneal inflammation after alkali burns.

In summary, this study demonstrates that exogenous netrin-4 inhibits HUVEC tube formation, proliferation, migration, and invasion, but promotes HUVEC apoptosis. In addition, netrin-4 inhibits and reverses neovascularization in alkali-burned corneas. Corneal neovascularization is a major cause of corneal blindness and a risk factor for rejection following allograft corneal transplantation. The multifunctional features of netrin-4 may shed new light on possible treatments for angiogenic diseases that affect the ocular surface, as well as other tissues. However, the underlying molecular mechanism is largely unknown. A futher study using siRNA silencing, inhibitor or mutant mice could be used in the future.

## Supporting Information

S1 Fig Effect of different doses of netrin-4 on NV area and inflammatory index induced by alkali-burn rat cornea. (A) 5000 ng/mL netrin-4 inhibited CNV area significantly compared with other groups on day 14 (** *p* < 0.01). (B) On the 7d, inflammatory index reduced significantly by 5000 ng/mL netrin-4 (** *p* < 0.01).
(TIF)
