# Supplementary figures and images for: Therapeutic Effects of Topical Netrin-4 Inhibits Corneal Neovascularization in Alkali-Burn Rats
Source: PLoS One. 2015 Apr 8;10(4):e0122951. doi: 10.1371/journal.pone.0122951 (PMC4390284; doi:10.1371/journal.pone.0122951)

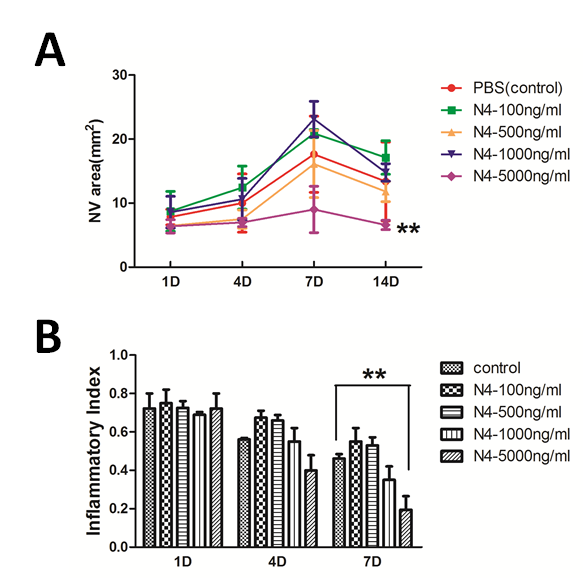

Supplement: S1 Fig — (A) 5000 ng/mL netrin-4 inhibited CNV area significantly compared with other groups on day 14 (** p < 0.01). (B) On the 7d, inflammatory index reduced significantly by 5000 ng/mL netrin-4 (** p < 0.01). (TIF) [file pone.0122951.s001.tif]
